# The comparative genomic analysis provides insights into the phylogeny and virulence of tick-borne encephalitis virus vaccine strain Senzhang

**DOI:** 10.1371/journal.pone.0273565

**Published:** 2022-08-26

**Authors:** Meng Zhang, Jingyong Tian, Hongying Li, Ming Cang

**Affiliations:** 1 State Key Laboratory of Reproductive Regulation and Breeding of Grassland Livestock, College of Life Sciences, Inner Mongolia University, Hohhot, People’s Republic of China; 2 Department of Pediatrics, Tongliao City General Hospital, Tongliao, Inner Mongolia, People’s Republic of China; International Centre for Genetic Engineering and Biotechnology, ITALY

## Abstract

Tick-borne encephalitis virus (TBEV) is one of the most dangerous tick-borne viral pathogens for humans. It can cause severe tick-borne encephalitis (TBE), multiple neurological complications, and death. The European subtype (TBEV-Eu), Siberian subtype (TBEV-Sib), and Far-Eastern subtype (TBEV-FE) are three main TBEV subtypes, causing varying clinical manifestations. Though TBEV-FE is the most virulent TBEV subtype, the degree of variation in the amino acid sequence of TBEV polyprotein is not high, leaving an issue without proper explanation. We performed phylogenic analysis on 243 TBEV strains and then took Senzhang strain as a query strain and representative strains of three major TBEV subtypes as reference strains to perform the comparative genomic analysis, including synteny analysis, SNP analysis, InDel analysis, and multiple sequence alignment of their envelope (E) proteins. The results demonstrated that insertions or deletions of large fragments occurred at the 3’ end but not at the 5’ end or in the CDS region of TBEV Senzhang strain. In addition, SNP sites are mainly located in the CDS region, with few SNP sites in the non-coding region. Our data highlighted the insertions or deletions of large fragments at the 3’ end and SNP sites in the CDS region as genomic properties of the TBEV Senzhang strain compared to representative strains with the main subtypes. These features are probably related to the virulence of the TBEV Senzhang strain and could be considered in future vaccine development and drug target screening for TBEV.

## Introduction

Tick-borne encephalitis virus (TBEV) is a tick-borne viral pathogen that can infect humans or livestock and is one of the most predominant, dangerous tick-borne pathogens for humans [1]. Most people infected with TBEV do not show significant symptoms, but those with symptoms usually present with clinical manifestations of the central nervous system. For example, meningoencephalitis, meningoencephalitis, and encephalitis, which are collectively referred to as tick-borne encephalitis (TBE), is a severe infectious disease that often results in long-term neurological complications, even lifelong, and can lead to death [2]. The morbidity and mortality of TBE vary depending on the subtypes of infected TBEV, which are the European subtype (TBEV-Eu), the Siberian subtype (TBEV-Sib), and the Far-Eastern subtype (TBEV-FE) [3]. Besides, two new TBEV subtypes have been reported: the Himalaya subtype and Baikalia subtype [3, 4]. In addition to causing a high frequency of neurological sequelae, the TBEV-FE can also cause up to 40% of mortality, compared with 10% for the TBEV-Eu and 2% for the TBEV-Sib [5].

Few TBEV-Eu infected patients present with clinical symptoms. For those symptomatic patients, the TBEV-Eu strains are characterized by causing a mild biphasic infection. In the first viremic phase, patients usually develop an influenza-like syndrome; in the second phase, patients exhibit neurological diseases with varying severity [2]. The TBEV-Sib strains tend to cause moderately severe clinical manifestations, which are mainly associated with a high incidence of non-paralytic febrile encephalitis [6]. In addition, some patients develop chronic progressive TBE, including the clinical presentation of Kozhevnikov’s epilepsy, Parkinson’s-like disease, and progressive neuritis, among others [1]. Chronic progressive TBE mainly affects children and working-age people [7]. The TBEV-FE strains cause the most severe central nervous system symptoms, often with acute onsets, such as sudden high fever, neurological damage, meningeal syndrome, and even paralysis [8]. Patients can have a severe clinical presentation of coma, focal encephalitis, meningoencephalitis, and myelitis, which often involve the brainstem and spinal cord [8, 9]. Moreover, recent serological evidence suggests that asymptomatic or nonspecific manifestations may be present in cases of infection with TBEV-FE strains [10], thus adding difficulty to the diagnosis and treatment of such cases and the vaccine development for TBEV-FE strains.

However, the degree of pathogenicity-related variation in the amino acid (AA) sequence of the TBEV polyprotein is not high: Sequence alignment identified two unique AA substitutions of the envelope (E) protein of a European subtype TBEV strain 93/783 as contributing to its increased pathogenicity compared to other TBEV strains of Siberian, Far-Eastern, and European subtypes [11]. Besides, it was reported that among TBEV strains causing the encephalitic form (Efd group), the febrile form (Ffd group), or the subclinical form (Sfd group) of diseases, only 17 AA substitutions were related to their variable pathogenicity for humans, and the AA variation authentically differed between TBEV groups [12]. Therefore, it is hypothesized that the magnitude of virulence within the same or among different TBEV subtypes may depend on a minimal number of AA residue alterations in the viral protein, and the variation of non-coding regions in the viral genome may also play a role. However, detailed investigations are lacking so far [13].

The mature TBEV particle is about 50 nm in diameter and has an envelope composed of membrane proteins and envelope proteins located under a lipid bilayer. TBEV has a single positive-stranded RNA genome, about 11kb long (10,405–11,103 nucleotides), with an open reading frame (ORF) encoding a polyprotein, which is cleaved into three structural proteins (C, M, and E) and seven non-structural proteins (NS1, NS2A, NS2B, NS3, NS4A, NS4B, and NS5) after co- and post-translational protease processing [14]. The envelope (E) protein acts as a viral surface glycoprotein that mediates receptor binding and viral fusion with endosomal membranes. Moreover, it is critical to activate protective immunity [15]. Thus, it is suggested that the E protein of TBEV could be tightly associated with virulence, which indicates TBEV’s ability to enter the target cells and establish a productive infection [16].

TBE has been endemic in the northeastern forests of China for a long time. To prevent TBEV infection and the prevalence of TBE, vaccines against TBE were developed based on TBEV Senzhang, Senhou, and CoΦ strains in China in 1953 [17]. After successive optimization to reduce adverse reactions such as allergic reactions, an inactivated vaccine with the Senzhang strain as the seed strain was finally established and widely used since the 1970s [17, 18]. Nevertheless, the efficacy of the acquired vaccine was undesirable [19]. Characterized as causing an encephalitic form of the disease after infection, the Senzhang strain has been discovered to present genomic and proteinic variation compared to some TEBV strains causing less severe disease forms or several other TEBV strains isolated in northeastern forests of China [12, 20]. We wondered whether a characteristic variation of the Senzhang strain exists compared to representative strains of TBEV-Eu, TBEV-Sib, and TBEV-FE. In this study, one TBEV strain sequenced in Northeast China (Senzhang strain, TBEV-FE) was integrated and analyzed with other representative strains reported by NCBI (including TBEV-Eu, TBEV-FE, and TBEV-Sib). Through comparative genomic analysis to screen potential candidate antigens and differences in gene functions and virulence evolution processes among different virus strains, we aimed to provide new ideas for vaccine development and associated disease control of TBEV Senzhang strain.

## Methods

### Retrieval and annotation of TBEV sequences

We retrieved the whole-genome sequences of all TBEV published up to date from NCBI, then screened out those with ambiguous or inconsistent sequence descriptions or incomplete sequences. A total of 243 TBEV whole-genome sequence entries were obtained (S1 Table). The NCBI registration number, isolate name, isolate country, isolation time, strain subtype, and protein registration number of each sequence entry were recorded and matched with the GenBank database and literature to reconfirm and supplement the information, especially the isolated country, isolation time, and strain subtype. However, there were 17 TBEV sequences for which the corresponding subtype information was not retrieved, with the GenBank acc. #: KX268728, MG243699, KU761567, KJ739731, KJ739730, KJ739729, LC440460, LC440459, LC171402, LC017693, LC017692, KJ744034, MN542364, KT224353, KT224352, EF469662, and KJ633033. "Clone" was marked in the "isolate country" column if the sequence was obtained from experiments other than the natural state.

### Phylogenetic analysis

The 243 TBEV whole-genome sequences were collated and saved as a fasta format file and then employed for clustering analysis with MEGA software (version 7.0), which is widely used for molecular evolution and genetic analysis nucleic acid, amino acid multi-sequence alignment, biological evolution analysis [21]. The dendrogram was constructed using the Neighbor-Joining (NJ) method. One thousand replications was set as the parameter for bootstrap analysis to estimate the stability of the construction process.

### Comparative genomic analysis

The whole-genome sequence of TBEV Senzhang strain (GenBank acc. #: JQ650523) was used as the query sequence, and the whole genome sequences of representative strains of three main TBEV subtypes as reference sequences, which are European vaccine strain Neudoerfl (European subtype) with GenBank acc. # U27495, Russian vaccine strain 205 (Far-Eastern subtype) with GenBank acc. # JX498939, and typical Siberian subtype Vasilchenko strain with GenBank acc. # L40361 for following comparative genomic analysis, including synteny analysis, single nucleotide polymorphism (SNP) analysis, insertion-deletion (InDel) analysis, multiple sequence alignment analysis, and analysis of the basic properties and structure of E protein amino acid residues.

#### Synteny analysis

The Senzhang strain sequence was used as the query sequence for two-by-two comparisons with three reference sequences. The synteny analysis between the two genomes can reveal the genome-wide positional relationships of different strain sequences, including information on rearrangement and replication within the genome, insertions, and deletions between genomes. MUMmer (Version 3.22) software was used to compare the query genome with the reference genome to determine the large-scale synteny between the genomes. Then, LASTZ (version 1.02.00) software was used to compare the regions to determine the local positional alignment and to find the regions of Translocation (Trans), Inversion (Inv), and Trans+Inv.

#### SNP analysis

The MUMmer software was used to compare the query sequence with the reference sequences globally to find out the sites that differed between the Senzhang strain sequence and the reference sequence and perform preliminary filtering to detect potential SNP sites. 100 nt on both sides of the SNP sites of the reference sequence were extracted, and then the extracted sequences were compared with the query sequence using BLAT software to verify the SNP sites. If the comparison length is less than 101 nt, it is considered an unreliable SNP and will be removed; if the comparison matches several times, it is considered an SNP in the duplicated region and will be removed. Then, the duplicated sequence region of the reference sequence is predicted by BLAST, TRF, and Repeatmask software, and the SNPs located in the duplicated region are filtered, and reliable SNPs are obtained at last.

#### InDel analysis

The query sequence was compared with the reference sequence using LASTZ software, and then the results were processed by axt_correction, axtSort, and axtBest programs to select the best comparison results and obtain preliminary InDel results. Then, 150 nt of upstream and downstream of the InDel site of the reference sequence were compared with the query sequence with BWA software and SamTools, and reliable InDel items were obtained after filtering.

#### Multiple sequence alignment of envelope protein E

The envelope protein E was identified as the feature region, and the corresponding intervals in query sequence and reference sequences were extracted, which were then input into Jalview 2.11.1.4 (http://www.jalview.org/getdown/release/) to perform the multiple sequence alignment.

### Analysis of fundamental properties of E protein

ProtParam, ProtScale, TMHMM Server, and NetPhos3.0 Server were used to analyze the physicochemical properties, hydrophobicity, transmembrane region prediction, and phosphorylation site prediction of the envelope protein E of the TBEV Senzhang strain, respectively. The physicochemical properties of the protein sequence were predicted using ProtParam (https://web.expasy.org/protparam/), including amino acid residue composition and content, relative molecular mass, isoelectric point, hydrophilicity index, and lipid coefficient, with an instability coefficient of less than 40; Using ProtScale (https://www.expasy.org/proscale/) to predict the hydrophobicity of the protein sequence, and the corresponding derived score determined the hydrophobicity of E protein; TMHMM Server was used to analyze the transmembrane helix region of the sequence to predict whether the protein is a transmembrane protein; NetPhos 3.0 Server was used to predict the glycosylation and phosphorylation sites in protein E and to analyze its biological function. The parameters of all software used in the above analysis were set as their default values.

### Epitopes and structure analysis of E protein

PSIPRED, Swiss-model, and DNAStar Protean were applied to analyze the secondary structure, tertiary structure, and protein surface antigenic epitope prediction of protein E of the TBEV Senzhang strain. PSIPRED (http://bioinf.cs.ucl.ac.uk/psipred/) for protein secondary structure prediction is based on a neural network algorithm that uses multiple sequence matching and extracts multiple sequences, protein structures, and evolutionary information from relevant databases to derive the secondary structure of the target protein. After predicting protein E secondary structure, the prediction results were displayed using PSIPRED and SOPMA software; Swiss-model (https://swissmodel.expasy.org/) is a protein structure homology-modeling server based on the NCBI database and was adopted to construct the 3D structure model of E protein for spatial structure visualization. DNAStar Protean software was used to analyze the surface epitopes of E protein by the Jameson-Wolf method to analyze the antigen activity.

## Results

### Phylogeny of 243 strains of TBEV

**Fig 1:**
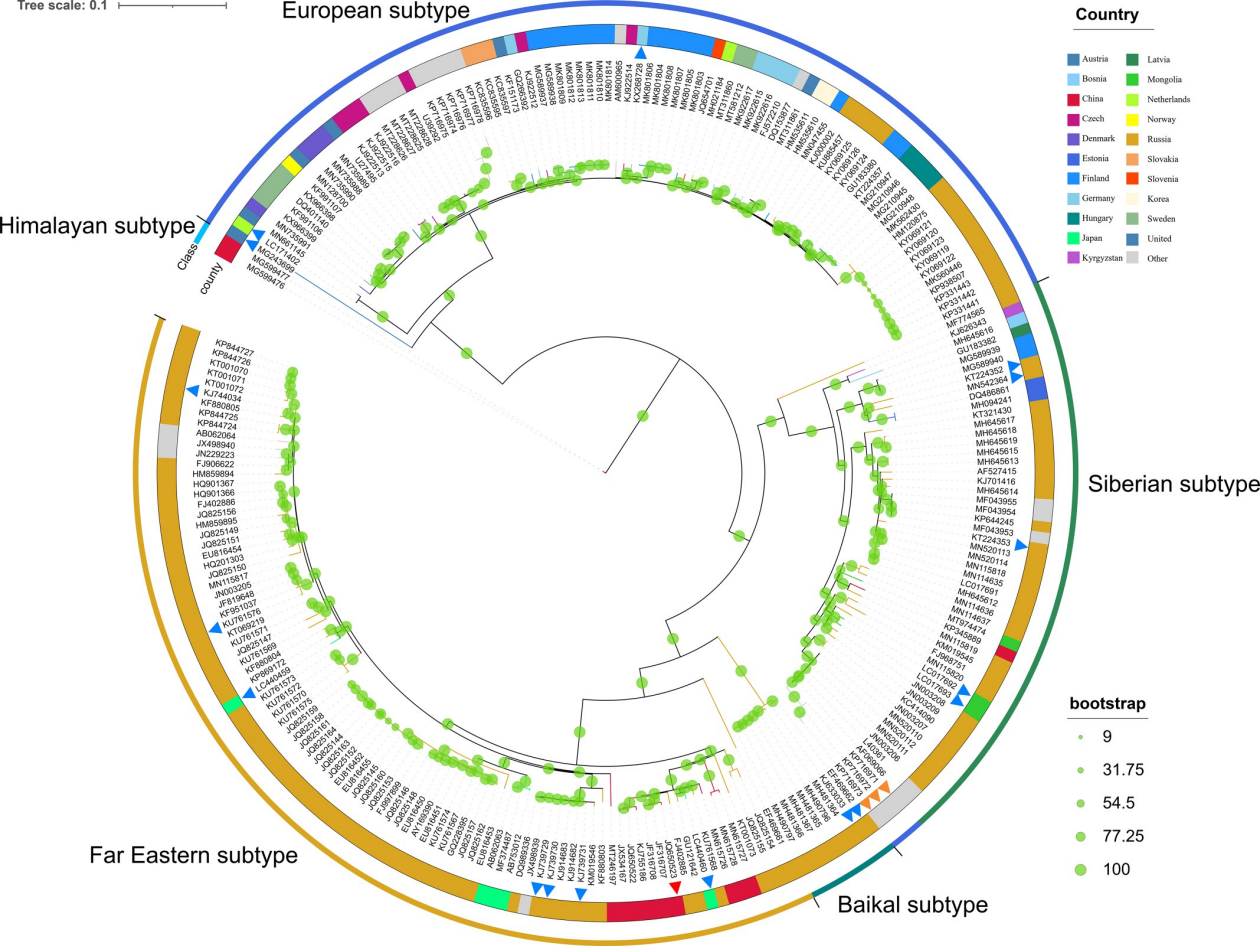
The phylogenic tree of 243 TBEV strains. The red triangle indicates the Senzhang strain; the orange triangles indicate three strains with subtypes inconsistent with the retrieved information, and the blue triangles indicate 17 strains without recorded subtypes but subtyped by the phylogenic analysis.

The whole-genome sequences of 243 TBEV strains were integrated to construct a phylogenic tree (Fig 1). These TBEV strains from more than 20 countries were classified into three major subtypes: the Far-Eastern subtype, the European subtype, and the Siberian subtype. Besides, two new subtypes, the Himalayan subtype and the Baikal subtype were also identified. These data were consistent with all currently reported TBEV subtypes. A total of 13 TBEV strains with whole-genome sequences were isolated and obtained in China, of which two strains were Himalayan subtypes, one strain was a Siberian subtype, and the rest all belonged to the Far-Eastern subtype. The Senzhang strain (JQ650523) was on the same branch as the two strains, JF316707 and JF316708, found in Mudanjiang, Heilongjiang Province, China, indicating their closest kinship and similar genetic evolution, which is consistent with the reported findings [19].

The results also showed three TBEV strains with subtypes inconsistent with the retrieved information, which were KP716971, KP716972, and KP716973. The records of the three strains showed that they should belong to European subtypes, but the phylogenic analysis suggested that they were more likely to be Siberian subtypes. In addition, 17 strains without recorded subtypes were subtyped by our analysis: the European subtype: KX268728, MG243699, LC171402; the Far-Eastern subtype: KU761567, KJ739731, KJ739730, KJ739729, LC440460, LC440459, KJ744034; the Siberian subtype: LC017693, LC017692, MN542364, KT224353, KT224352; and the Baikalia subtype: EF469662, KJ633033. However, this theoretical classification should not be considered final results and needs further verification.

### Synteny analysis showed variation at the 3’ end of the Senzhang strain

**Fig 2:**
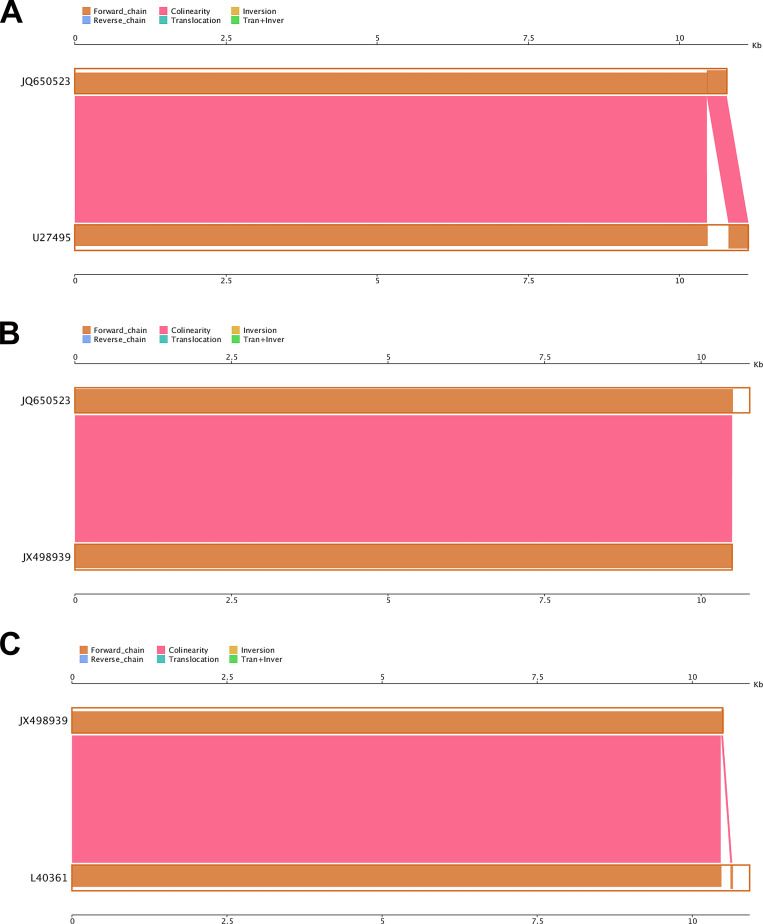
TBEV genome-wide synteny analysis. A: Comparison of Senzhang strain and Neudoerfl strain; B: Comparison of Senzhang strain and 205 strain; C: Comparison of Senzhang and Vasilchenko strain. Query sequence: Senzhang strain, JQ650523; reference sequences: Neudoerfl strain, U27495; 205 strain, JX498939; Vasilchenko strain, L40361.

Genome-wide synteny analysis of the Senzhang strain and three reference strains (Neudoerfl strain for European subtype [22], 205 strain for Far-Eastern subtype [23, 24], and Vasilchenko strain for Siberian subtype [12, 25]) showed that the Senzhang strain had high synteny with the other three subtypes. However, there was significant variation at the 3’ end of the genome sequence. Compared with the Neudoerfl strain, the Senzhang strain had a significant deletion at the 3’ end (Fig 2A); compared with the 205 strain, the Senzhang strain had an additional sequence at the 3’ end (Fig 2B); and compared with Vasilchenko strain, Senzhang strain also had a significant deletion at the 3’ end (Fig 2C). These results suggest that large insertions and deletions occur mainly at the end of the Senzhang strain genome compared to the representative strains of three major TBEV subtypes. The coverages indicated high similarity between the Senzhang strain and reference strains, with 96.8%, 97.36%, and 98.69% similarity to Neudoerfl, 205, and Vasilchenko strains, respectively greater than 96%. Additionally, in terms of the number of inserted and missing bases, the Senzhang strain differed the most from the Neudoerfl strain, with 356 nt missing, with 285 nt increased compared with 205 strain, and with 143 nt missing compared with the Vasilchenko strain, which showed that the probability of variation at the 3’ end of different TBEV subtypes was high. In contrast, the 5’ end and the CDS (Coding Sequence) region were highly conserved.

### SNP and InDel analysis revealed abundant mutations in the CDS region of the Senzhang strain

**Fig 3:**
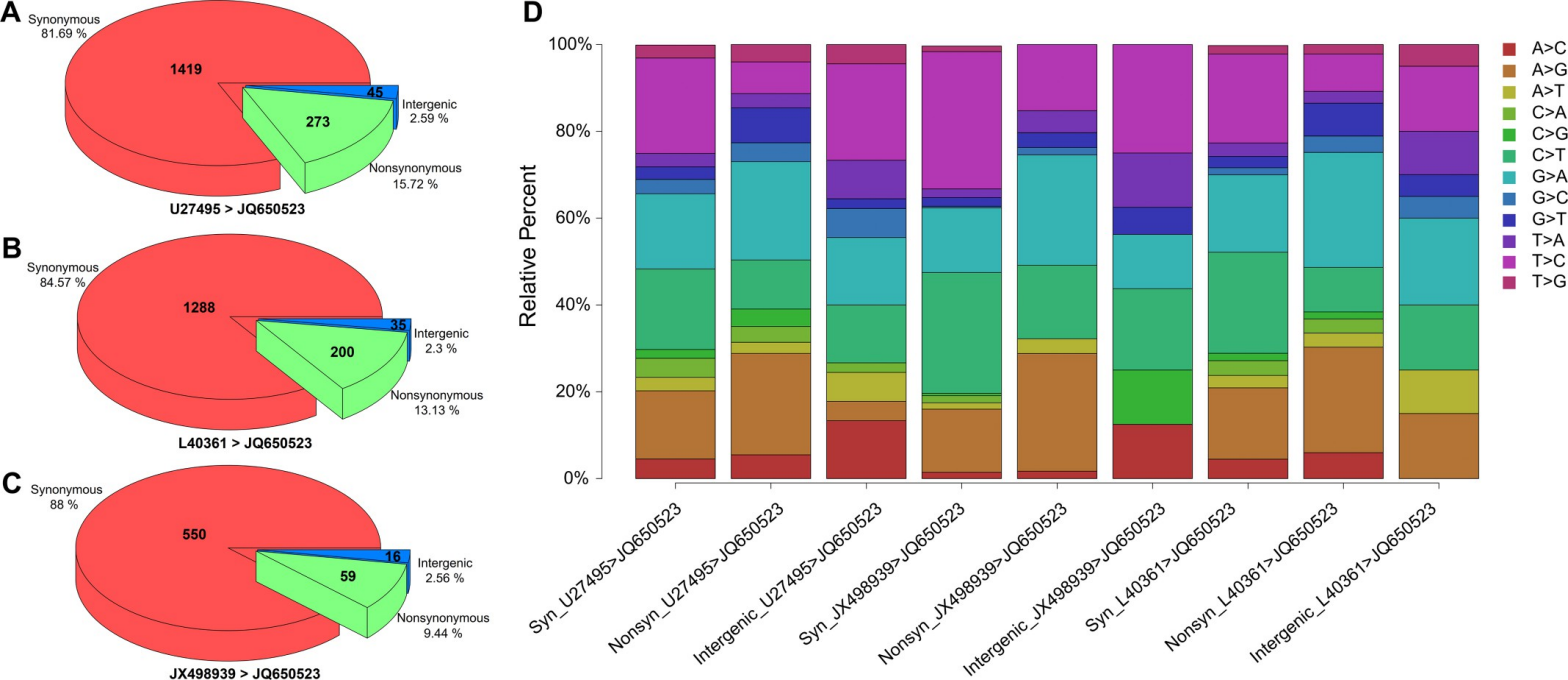
SNPs of Senzhang strain compared to reference strains. A: Distribution of SNP types of Senzhang strain compared to Neudoerfl strain. B: Distribution of SNP types of Senzhang strain compared to Vasilchenko strain. C: Distribution of SNP types of Senzhang strain compared to 205 strain. For A to C graph, the amount of SNPs was marked in the corresponding part of the pie diagram. D: Relative percentage of specific base substitution for SNPs of Senzhang strain compared to reference strains. Syn: Synonymous; Nonsyn: Nonsynonymous.

The results of SNP analysis include synonymous mutations of the start codon (Start_syn), synonymous mutations of the stop codon (Stop_syn), nonsynonymous mutations of the start codon (Start_nonsyn), nonsynonymous mutations of the stop codon (Stop_nonsyn), intragenic synonymous mutations (Synonymous), intragenic nonsynonymous mutations (Nonsynonymous), and mutations in the intergenic region (Intergenic). The analysis showed that the Senzhang strain had the most abundant variation compared to the European subtype Neudoerfl strain (U27495) (Fig 3A), slightly less abundant variation compared to the Siberian subtype Vasilchenko strain (L40361) (Fig 3B), and much less variation compared to Far-Eastern subtype 205 strain (JX498939) (Fig 3C) on total SNPs, Nonsynonymous SNPs, and Intergenic SNPs. No SNP was identified as Start_syn, Stop_syn, Start_nonsyn, or Stop_nonsyn. Statistics on specific base substitution of SNPs showed that Neudoerfl strain and Vasilchenko strain shared similar patterns, away from 205 strain (Fig 3D). The SNP sites were mainly in the CDS region, with few SNP sites in the non-coding region. InDel analysis revealed that only one fragment deletion (49–51, 2 nt) and one fragment insertion (10449–10454, 6 nt) were found in the Senzhang strain compared with the 205 strain, and no insertion or deletion was found compared with the other two strains. We also analyzed SNP sites in the CDS region of less pathogenic Neudoerfl that are not presented in more pathogenic Hypr strain compared to the Senzhang strain. We uncovered 130 SNP sites, 13 of which are nonsynonymous, and the rest are synonymous (S2 Table).

### Basic properties, epitope, and structural features of E protein of Senzhang strain

The E protein of the Senzhang strain is located in the whole genome from 970 nt to 2457 nt, totaling 1488 nt, encoding 496 amino acids (AA), which is located at the position of polyprotein 281–776 AA. The molecular weight of E protein is 53684.59. The theoretical isoelectric point (PI) is 8.17, and the total number of atoms is 7534. Among the encoded amino acids, glycine (Gly) has the highest content, accounting for 10.9% of the whole AA sequence, and tryptophan (Trp) and tyrosine (Tyr) have the lowest content, accounting for 2.00% of the AA sequence.

**Fig 4:**
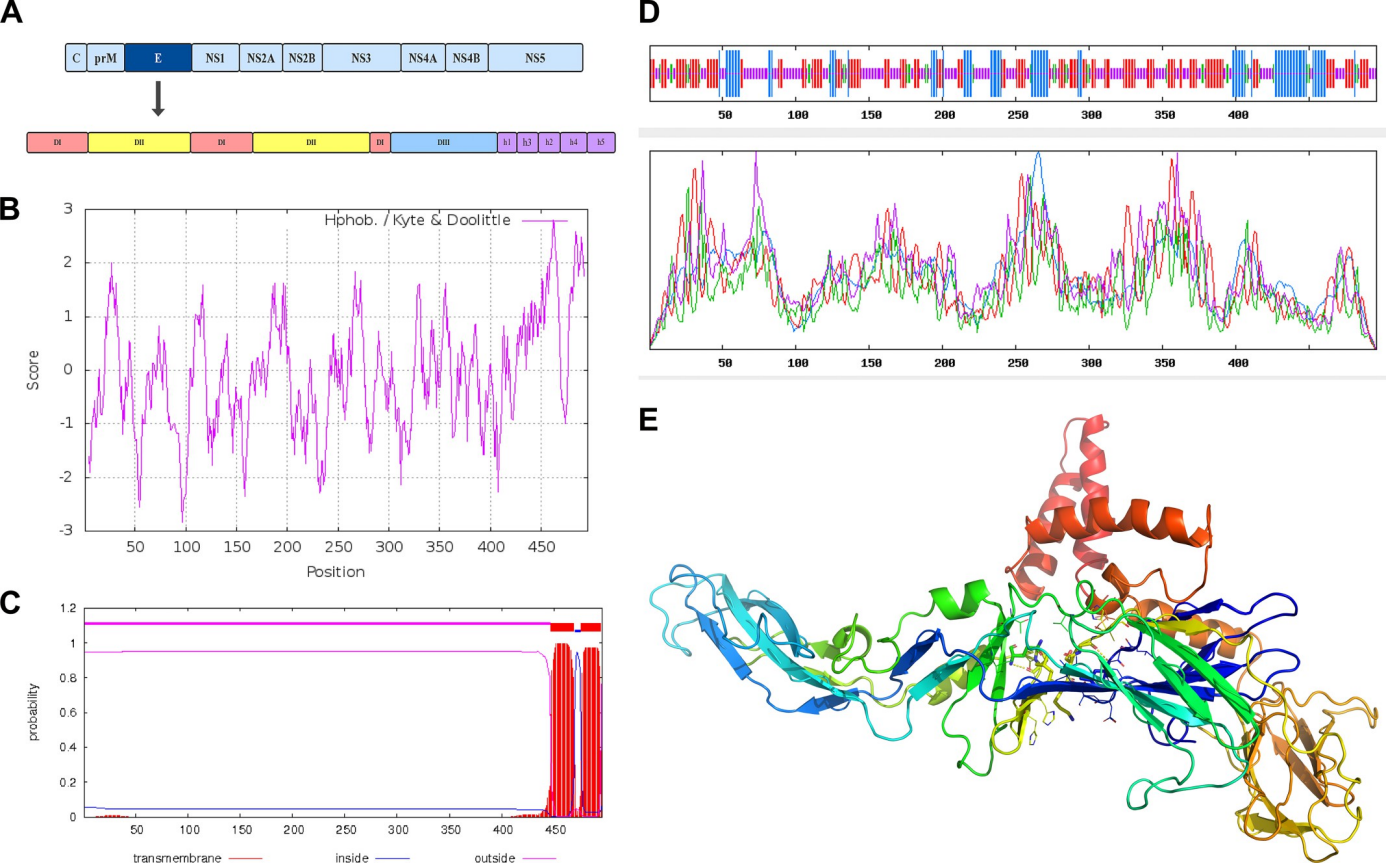
Physicochemical properties and structural features of E protein of Senzhang strain. A: Schematic diagram of E protein structural domains. B: Hydrophobicity evaluation of E protein. C: Predicted transmembrane region of E protein. D: Predicted secondary structure of E protein. E: A tertiary structural model of E protein.

The E protein contains four structural domains (Fig 4A), of which Domain I (DI) has 122 AA residues divided into three fragments, 1–51 AA, 137–189 AA, and 285–302 AA, respectively. Two sequences in the middle of the three fragments form two loop structures as Domain II (DII), at the specific positions of 52–136 AA and 190–284 AA, with a highly conserved viral fusion peptide (98–110 AA) at the top, and this region is rich in glycine and hydrophobic, which is conducive to fusion with the cell membrane. The 303–400 AA region is Domain III (DIII). DI—DIII accounts for about 80% of the E protein region or 20% of the carboxyl-terminal region. The region of 401–496 AA is Domain IV (DIV). DIV contains two α-helices h1 (401 to 413 AA) and h2 (431 to 449 AA), with a conserved element h3 (414 to 430 AA) between them, forming the stem region. DIV also contains two transmembrane α-helices h4 (450 to 472 AA) and h5 (473 to 496 AA), forming the anchor region. The hydrophobicity analysis of the E protein revealed that E protein has a maximum value of 2.8 at the 462nd amino acid site (Fig 4B), which is strongly hydrophobic and facilitates the fusion of the E protein with the cell membrane.

The prediction of the transmembrane structural domain of E protein revealed that the protein has two transmembrane regions between 450 and 496 AA (Fig 4C). The antigenic epitopes of E protein were predicted, and 21 B-cell antigenic epitopes were found to be present with a uniform distribution. The analyses of E protein’s predicted secondary (Fig 4D) and tertiary structures (Fig 4E) showed that E protein mainly consisted of β-fold and helical structures. The tertiary structure modeling revealed that the structural characteristics of E protein were consistent with the predicted secondary structure. The above predicted and analyzed results were consistent with published structures of TBE virus E proteins [26].

### Multiple sequence alignment of E proteins of TBEV

**Fig 5:**
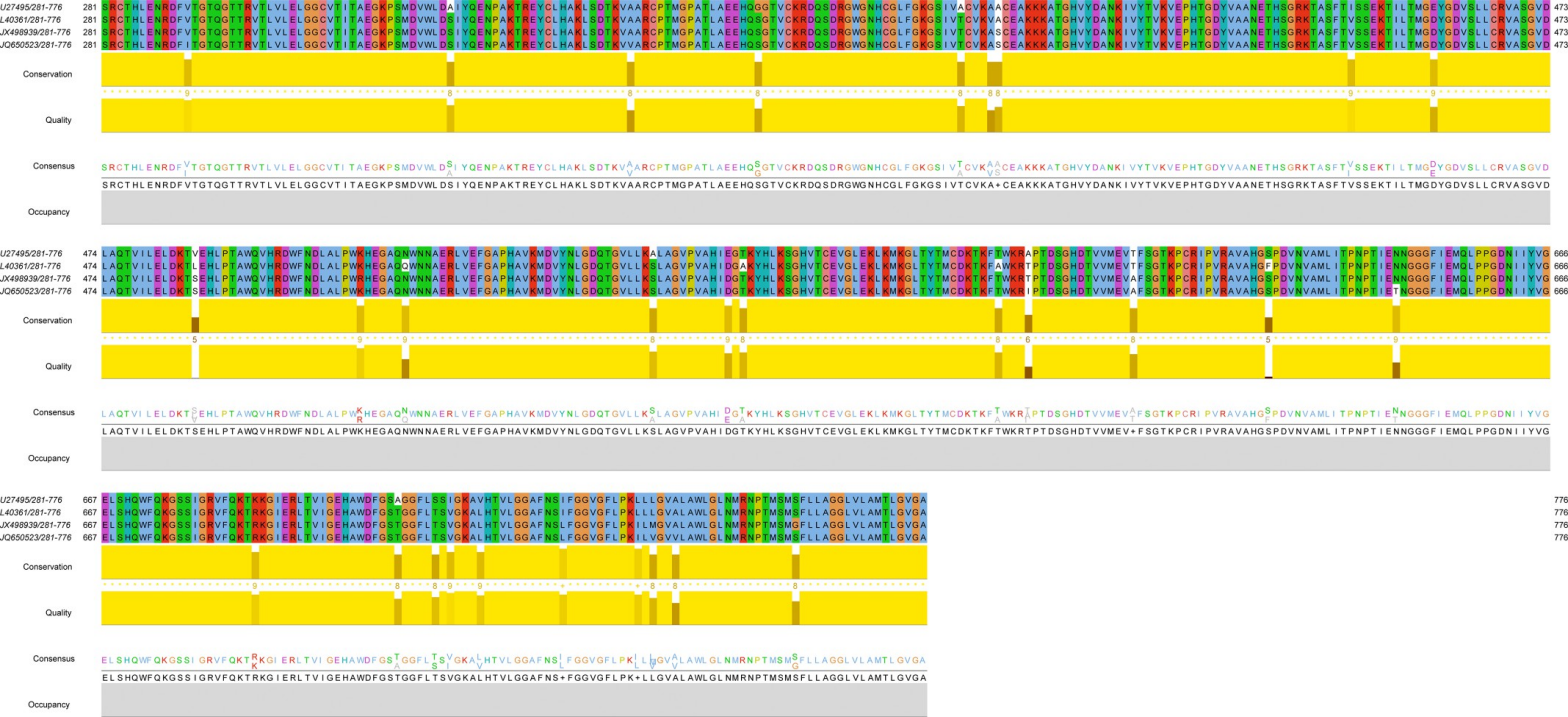
Multiple AA sequence alignment of TBEV E proteins.

The AA sequence analysis and AA multiple sequence comparison differential analysis of the E protein of the Senzhang strain showed that there were 30 sites with varying AA types among 496 AAs in the E protein (Fig 5). With four structural domains of the E protein for variation statistics (Table 1), the results showed that four aligned TBEV strains had AA sequence inconsistencies at four sites in E protein DI, 11 sites in DII, five sites in DIII, and ten sites in DIV. From the comparison of the Senzhang strain with reference strains, there were 23 sites of AA alterations when compared with the Neudoerfl strain (DI: I12V, S47A, V167I, D178E; DII: V71A, S88G, T115A, S120A, S206V, S267A, D277E; DIII: I317A, A331T, T366N; DIV: R407K, T426A, T431S, V433I, L437V, L448I, I458L, V460L, V463A), with 95.36% homology; there were 16 sites of AA alterations compared with the Vasilchenko strain (DI: I12V; DII: V71A, A119V, S120A, S206L, N234Q, T279A; DIII: T313A, I317T, A331T, S349F, T366N; DIV: L448I, I458L, V460L, V463A), with 96.77% homology; and there were eight sites of AA alterations compared with the 205 strain (DI: I12V; DII: V71A, K228R; DIII: I317T, T366N; DIV: V460M, V463A, S479G), with 98.39% homology. Besides, the fewest AA substitution sites and the highest homology of E protein were observed in the comparison of the TBEV-Eu Neudoerfl strain with the TBEV-Sib Vasilchenko strain.

**Table 1:** AA variation statistics of E protein of Senzhang strain and reference strains.

AA sites	European subtype Neudoerfl strain U27495	Siberian subtype Vasilchenko strain L40361	Far Eastern subtype 205 strain JX498939	Far Eastern subtype Senzhang strain JQ650523
DI-12	V	V	V	I
DI-47	A	S	S	S
DI-167	I	V	V	V
DI-178	E	D	D	D
DII-71	A	A	A	V
DII-88	G	S	S	S
DII-115	A	T	T	T
DII-119	A	V	A	A
DII-120	A	A	S	S
DII-206	V	L	S	S
DII-228	K	K	R	K
DII-234	N	Q	N	N
DII-267	A	S	S	S
DII-277	E	D	D	D
DII-279	T	A	T	T
DIII-313	T	A	T	T
DIII-317	A	T	T	I
DIII-331	T	T	A	A
DIII-349	S	F	S	S
DIII-366	N	N	N	T
DIV-407	K	R	R	R
DIV-426	A	T	T	T
DIV-431	S	T	T	T
DIV-433	I	V	V	V
DIV-437	V	L	L	L
DIV-448	I	I	L	L
DIV-458	L	L	I	I
DIV-460	L	L	M	V
DIV-463	A	A	A	V
DIV-479	S	S	G	S

## Discussion

The three major TBEV subtypes, along with other critical human pathogens such as Japanese encephalitis virus (JEV) and West Nile virus (WNV), belong to the family *Flaviviridae*, genus *Flavivirus* [27, 28]. Many flaviviruses are transmitted by mosquitoes, such as JEV, and have been extensively studied because of the widespread attention given to the significant risks they pose to human health. Tick-borne flavivirus pathogens, such as TBEV, lack sufficient attention compared to mosquito-borne flavivirus pathogens. This situation has led to a lack of understanding of many TBEV properties. In particular, knowledge of the genetic and structural details of TBEV infection has been primarily based on inference from the more well-characterized mosquito-borne flaviviruses [29]. So, it is clear that despite the significant progress made in recent years in the field of flaviviruses, many aspects of the molecular biology of TBEV are still poorly characterized. The methodology proving that TBEV pathogenicity is associated with its genetic factors has been well developed in recent years. For example, generating clones of TBEV with specific poly(A) tracts or mutations in certain genomic regions which might contribute to virulence determinants [30], or generating infectious cDNA clones of TBEV with reverse genetics systems can provide useful platforms to investigate the genetic determinants of TBEV virulence [31]. Besides, genomic sequencing and genetic and phenotypic comparison can also provide important information [32]. We performed a whole-genome-wide comparative genomic analysis of the TBEV Senzhang strain with the major three TBEV subtypes as reference strains to reveal detailed genomic variation among different virus strains and the possible evolution of virulence.

Interestingly, we identified 17 TBEV strains without recorded subtype information and 3 TBEV strains with subtypes inconsistent with the records in the phylogenic analysis. However, we retrieved relevant literature and found that subtypes were indicated for 5 TBEV strains without public recorded subtypes: KX268728, European subtype [33]; KJ739731, LC440459, LC440460, Far-Eastern subtype [34]; KJ744034, Far-Eastern subtype [35]. These data were consistent with our phylogenic analysis. We thought that it might result from increasingly sophisticated data from databases and continuous publication of literature on TBEV and our growing knowledge of the subject. Perhaps we provide a complete TEBV evolutionary tree, which could suggest further exploration of TEBV subtype delineation and genome evolution.

The synergy analysis suggested a greater probability of variation at the 3’ end and obvious conservation at the 5’ end and in the CDS region of sequences of TBEV Senzhang strain. It has been reported that alterations of the 3’-untranslated region can change its conformational structure, which could be associated with the replication efficiency and virulence of TBEV [36, 37]. Our data on the TBEV Senzhang strain is consistent with previous findings, suggesting a possible connection of variability of the 3’-untranslated region to the virulence of the TBEV Senzhang strain. The SNP and InDel data showed that SNP sites mainly occurred in the CDS region, with few in the non-coding region. Besides, insertions or deletions of small fragments were rarely observed. In addition, the number of bases in the CDS region of different TBEV subtypes is remarkably conserved, which is 10245 nt, encoding a polyprotein composed of 3414 AAs, while the number of bases at the start site of their coding regions is very little different. Therefore, we speculated that SNP variation causing changes of encoded AAs was another critical factor that affected the corresponding protein functions and further formed the TBEV Senzhang strain with more virulence.

Additionally, we uncovered 130 SNP sites in the CDS region of less pathogenic Neudoerfl that are not presented in more pathogenic Hypr strain compared to Senzhang strain. Although these SNP sites represent only a small fraction of that in the CDS region of the Neudoerfl strain compared to the Senzhang strain (1419 vs. 117 synonymous mutations and 273 vs. 13 nonsynonymous mutations), we think that the possibility that this small fraction of SNP sites contributes to the virulence difference between the Neudoerfl and Hypr strains cannot be dismissed entirely. In addition, another possibility that should not be ignored is that critical SNP sites in the CDS region need to accumulate to a certain amount to have an effect on virulence. However, these need further experimental confirmation.

The severity of symptoms in TBE patients is related to the TBEV subtypes. At the same time, the E protein of the TBEV is an essential component of the viral surface structure, contains an antigenic determinant, and is involved in viral attachment, membrane fusion, immune response, and pathogenesis. Thus, the E protein of TBEV is thought to be associated with virulence [16].

The AA variation of E protein of the Senzhang strain and reference strains showed that the Senzhang strain had only three unique substituted amino acid sites (DI-12, DII-71, DIII-366) compared to three reference strains, and the Senzhang strain, together with the 205 strain had five substituted amino acid sites (DII-120, DII-206, DIII-331, DIV-448, DIV-458) compared to the other two subtypes of strains. DI-12 is located in the first fragment region of the first structural domain, which is mainly a protein center for maintaining stability and has little relationship with the biological properties of the virus [38]. DII-71, DII-120, and DII-206 are located in the second structural domain of the E protein, which is involved in TBEV binding to host cell membrane receptors and TBEV attachment, with protein conformational changes during cell membrane fusion. The specific AA substitution in this region may affect TBEV attachment, causing a decrease in viral titers, affecting the cell membrane fusion process, and causing a decrease in neurovirulence [39]. DIII-331 and DIII-336 are located in the third structural domain of the E protein, which is the primary antigenic region of the E protein and a critical region for TBEV virulence. AA changes in this region can affect the receptor binding site, thus reducing neuroinvasiveness. DIV-448 and DIV-458 are located in the fourth structural domain of E protein, with DIV-448 in the stem region and DIV-458 in the transmembrane region, which can be stabilized with the M protein to form an E-M-M-E heterotetrameric structure and involved in conformational changes at a low pH value. So AA changes in this region could affect viral invading and replication, especially playing a vital role in releasing nucleocapsid into the host cytoplasm [40].

## Conclusion

This study highlighted the insertions or deletions of large fragments at the 3’ end and SNP sites in the CDS region as genomic properties of the TBEV Senzhang strain compared to representative strains with the main subtypes. These features are probably related to the virulence of TBEV Senzhang strain and could be considered in future vaccine development and drug target screening for TBEV.

## Supporting information

S1 Table A total of 243 TBEV whole-genome sequence entries. (PDF)

S2 Table SNP sites in the CDS region of Neudoerfl strain that are not presented in Hypr strain compared to Senzhang strain. (PDF)
