# Supplementary material for: The comparative genomic analysis provides insights into the phylogeny and virulence of tick-borne encephalitis virus vaccine strain Senzhang
Source: PLoS One. 2022 Aug 26;17(8):e0273565. doi: 10.1371/journal.pone.0273565 (PMC9417034; doi:10.1371/journal.pone.0273565)
Supplement: S1 Table — (PDF) [file pone.0273565.s001.pdf]

ACCESSION	Isolate/Strain	Country	Collection Year	Class	ProtID
KX268728	MucAr-HB-171/11	Germany	2011	-	ANN44512
MG243699	N5-17/chamois/Austria/2017	Austria	2017	-	AZL96748
KU761567	Golubnichiy	Russia	1958	-	AMP18878
KJ739731	Tomsk-M83	Russia	2006	-	AIG24412
KJ739730	Tomsk-K6	Russia	2008	-	AIG24411
KJ739729	Novosibirsk-L2008	Russia	2008	-	AIG24410
LC440460	Nanporo-18-44	Japan	2018	-	BBH84734
LC440459	Sapporo-17-Io1	Japan	2017	-	BBH84733
LC171402	NL	Netherlands	2015	-	BAV60898
LC017693	MGL-Selenge-13-14	Mongolia	2014	-	BAQ25431
LC017692	MGL-Selenge-13-12	Mongolia	2014	-	BAQ25430
KJ744034	Malishevo	Russia	1978	-	AII96827
MN542364	Rus/Ix_persulcatus/Karelia/2/2018	Russia	2018	-	QPD01617
KT224353	LEIV-10133Al	Russia	1984	-	ALP82433
KT224352	LEIV-13652Ar	Russia	1984	-	ALP82432
EF469662	886-84	Russia	1984	-	ABS00285
KJ633033	886-84	Russia	1984	-	AIB53034
MH490797	3094-18_10	Russia	2010	Baikalia subtype	QBH22572
MH490796	43-99	Russia	1999	Baikalia subtype	QBH22571
MH481367	3094-9_10	Russia	2010	Baikalia subtype	QAB47429
MH481366	3033-1_10	Russia	2010	Baikalia subtype	QAB47428
MH481365	3094-29_10	Russia	2010	Baikalia subtype	QAB47427
MH481364	110-01	Russia	2001	Baikalia subtype	QAB47426
U27495	Neudoerfl	Austria	1971	European subtype	AAA86870
AM600965	K23	Germany	-	European subtype	CAM82856
KP938507	Sorex 18-10	Russia	2010	European subtype	AKE50910
KP331443	IrkutskBR_1456-09	Russia	2009	European subtype	AKE50895
KP331442	IrkutskBR_1434-09	Russia	2009	European subtype	AKE50894
KP331441	IrkutskBR_99-08	Russia	2008	European subtype	AKE50893
KF151173	A104	Austria	1990	European subtype	AGP05331
HM535611	KrM 93	South	2006	European subtype	ADY80020
HM535610	KrM 213	South	2006	European subtype	ADY80019
MK560446	172-68	Russia	1968	European subtype	QIH04836
MT228628	40 PS	-	2020	European subtype	QOI58739
MT228627	parental_Hypr_strain_0	-	2020	European subtype	QOI58738
MT228626	LT IRE	-	2020	European subtype	QOI58737
MT228625	40 IRE	-	2020	European subtype	QOI58736
MT581212	93/783	Sweden	2019	European subtype	QOH98450
MN661145	UK-Hampshire 2019	United	2019	European subtype	QGI57862
MN735991	DEN09_Tokkekoeb	Denmark	2009	European subtype	QGX86230
MN735990	DEN19_S4A_Tisvilde	Denmark	2019	European subtype	QGX86229
MN735989	DEN19_S3_Tisvilde	Denmark	2019	European subtype	QGX86228
MN735988	DEN19_S3A_Tisvilde	Denmark	2019	European subtype	QGX86227
MN128700	UK-Thetford Forest 2018	United	2018	European subtype	QGA31138
MG589938	Kuutsalo-14_Ixodes_ricinus_Finland-2017	Finland	2017	European subtype	AWC08512
MG589937	Kuutsalo_Human_Cerebellum_Finland-2015	Finland	2015	European subtype	AWC08511
KX966399	JP-554	Sweden	2008	European subtype	APR62634
KX966398	JP-296	Sweden	2008	European subtype	APR62633
KP716978	Hypr[Vs_str]	Clone	2015	European subtype	AKP16371
KP716977	Hypr[Vs_prM-E]	Clone	2015	European subtype	AKP16370
KP716976	Hypr[Vs_E]	Clone	2015	European subtype	AKP16369
KP716975	Hypr_IC_[short_3'UTR]	Clone	2015	European subtype	AKP16368
KP716974	Hypr_IC	Clone	2015	European subtype	AKP16367
KP716973	Vs[Hypr_str]	Clone	2015	European subtype	AKP16366
KP716972	Vs[Hypr_prM-E]	Clone	2015	European subtype	AKP16365
KP716971	Vs[Hypr_E]	Clone	2015	European subtype	AKP16364
KJ922516	Vlasaty	Czech	1953	European subtype	AIL83864
KJ922515	Tobrman	Czech	1953	European subtype	AIL83863
KJ922514	Skrivanek	Czech	1953	European subtype	AIL83862
KJ922513	Petracova	Czech	1953	European subtype	AIL83861
KJ922512	Kubinova	Czech	1953	European subtype	AIL83860
KC835597	CGl223	Slovakia	1990	European subtype	AHF27217
KC835596	285	Slovakia	1990	European subtype	AHF27216

KC835595	114	Slovakia	1980	European subtype	AHF27215
DQ401140	Toro-2003	Sweden	2003	European subtype	ABD62793
U39292	Hypr	Czech	1953	European subtype	AAB53095
GQ266392	AS33	Germany	2005	European subtype	ADE22271
FJ572210	Salem	Germany	2006	European subtype	ACL97686
MT311861	Hochosterwitz	Austria	1971	European subtype	QLF98638
MT311860	1993/783	Sweden	1993	European subtype	QLF98637
MK922617	Rauher_BuschP19_S40，isolate="P0"	Germany	2018	European subtype	QDA01875
MK922616	HB_IF06_8040，P0	Germany	2018	European subtype	QDA01874
MK922615	HB_IF06_8033，P0	Germany	2018	European subtype	QDA01873
MH021184	NL/UH 2016	Netherlands	2016	European subtype	AWX59735
KY069126	Zmeinogorsk-9	Russia	1986	European subtype	AQY16004
KY069125	Zmeinogorsk-5	Russia	1986	European subtype	AQY16003
KY069124	Zmeinogorsk-1	Russia	1986	European subtype	AQY16002
KY069123	126-71	Russia	1971	European subtype	AQY16001
KY069122	262-74	Russia	1974	European subtype	AQY16000
KY069121	163-74	Russia	1974	European subtype	AQY15999
KY069120	118-71	Russia	1971	European subtype	AQY15998
KY069119	1G-98	Russia	1998	European subtype	AQY15997
KT224357	Kumlinge	Finland	1959	European subtype	ALP82437
KF991107	Mandal-2009	Norway	2009	European subtype	AHL20222
KF991106	Saringe-2009	Sweden	2009	European subtype	AHL20221
JQ654701	Ljubljana I	Slovenia	1992	European subtype	AFI49403
HM120875	84.2	Russia	2010	European subtype	ADM63091
DQ153877	temperature-resistant variant of strain 263	Clone	2005	European subtype	AAZ80455
MK562430	214-67	Russia	1967	European subtype	QIH04837
MK801813	K15-Espoo-Finland-2018	Finland	2018	European subtype	QCZ35635
MK801809	E266-Espoo-Finland-2017	Finland	2017	European subtype	QCZ35631
MK801808	Sipoo-23-Finland-2013	Finland	2013	European subtype	QCZ35630
MK801806	Sipoo-12-Finland-2013	Finland	2013	European subtype	QCZ35628
MK801805	Sipoo-8-Finland-2013	Finland	2013	European subtype	QCZ35627
MK801804	Sipoo-4-Finland-2013	Finland	2013	European subtype	QCZ35626
MK801803	Isosaari-5-Finland-2005	Finland	2005	European subtype	QCZ35625
MG210948	KEM-168	Hungary	2016	European subtype	AWH63451
MG210947	KEM-127	Hungary	2012	European subtype	AWH63450
MG210946	KEM-125	Hungary	2012	European subtype	AWH63449
MG210945	KEM-118	Hungary	2011	European subtype	AWH63448
KU885457	Absettarov	Russia	1951	European subtype	AMQ49166
KJ000002	Absettarov	Russia	1951	European subtype	AHM02467
MN047455	A10-Jollas-Finland-2016	Finland	2016	European subtype	QHN12651
MK801814	K16-Espoo-Finland-2018	Finland	2018	European subtype	QCZ35636
MK801812	K14-Espoo-Finland-2018	Finland	2018	European subtype	QCZ35634
MK801811	K13-Espoo-Finland-2018	Finland	2018	European subtype	QCZ35633
MK801810	K12-Espoo-Finland-2018	Finland	2018	European subtype	QCZ35632
MK801807	Sipoo-22-Finland-2013	Finland	2013	European subtype	QCZ35629
JN003209	Irkutsk-12	Russia	2010	Siberian subtype	AEQ77280
JN003208	Cht-22	Russia	2002	Siberian subtype	AEQ77279
JN003207	Cht-653	Russia	1995	Siberian subtype	AEQ77278
JN003206	Aina	Russia	1963	Siberian subtype	AEQ77277
MH645619	TBEV-2922	Russia	2012	Siberian subtype	AZS54291
MH645618	TBEV-2836	Russia	2012	Siberian subtype	AZS54290
MH645616	Bosnia-3	Bosnia	2000	Siberian subtype	AZS54288
MH645614	HimDym-6	Russia	1986	Siberian subtype	AZS54286
MH645613	Baikal-3	Russia	1986	Siberian subtype	AZS54285
MH094241	Ek-328c	Estonia	2018	Siberian subtype	AZI76174
FJ968751	Kolarovo-2008	Russia	2008	Siberian subtype	ACN42746
MN520114	253	Russia	1963	Siberian subtype	QRV07462
MN520113	562	Russia	1960	Siberian subtype	QRV07461
MN520112	210	Russia	1963	Siberian subtype	QRV07460
MN520111	206	Russia	1963	Siberian subtype	QRV07459
MN520110	506	Russia	1960	Siberian subtype	QRV07458
MN115820	518-66	Russia	1966	Siberian subtype	QDJ94242
MN115819	163-64	Russia	1964	Siberian subtype	QDJ94241
MN115818	41-65	Russia	1965	Siberian subtype	QDJ94240

MG589940	Kotka-18_Ixodes_ricinus_Finland-2011	Finland	2011	Siberian subtype	AWC08514
MG589939	uutsalo_2_Human_Cerebellum_Finland-201	Finland	2017	Siberian subtype	AWC08513
MF774565	TBEV-2871	Russia	2012	Siberian subtype	ATP62001
MF043955	C11-13-NEU	Clone	2017	Siberian subtype	ASG81457
MF043954	C11-13-293	Clone	2017	Siberian subtype	ASG81456
MF043953	C11-13PAK	Clone	2017	Siberian subtype	ASG81455
KP644245	C11-13	Russia	2013	Siberian subtype	AKL71380
KM019545	Tomsk-PT122	Russia	2015	Siberian subtype	AIL33471
KC414090	Zabaikalye 11-99	Russia	1999	Siberian subtype	AGI05090
AF069066	Vasilchenko	Clone	1998	Siberian subtype	AAD34205
KT321430	Konst-14	Russia	2014	Siberian subtype	AMD82540
KP345889	Sib-XJ-X5	China	2014	Siberian subtype	AKL59775
GU183382	Latvia-1-96	Latvia	1996	Siberian subtype	ADQ00971
DQ486861	EK-328	Estonia	1972	Siberian subtype	ABF46836
MT974474	92M	Mongolia	2004	Siberian subtype	QQR34430
MN114635	TSA-18	Russia	2018	Siberian subtype	QMS55021
MN114637	1827-18	Russia	2018	Siberian subtype	QMS55023
MN114636	1512-18	Russia	2018	Siberian subtype	QMS55022
KJ701416	Lesopark 11	Russia	1986	Siberian subtype	AIB53035
KJ626343	Buzuuchuk	USSR	1986	Siberian subtype	AIB53033
L40361	Vasilchenko	-	1982	Siberian subtype	AAF82240
LC017691	IR99-22f7	Russia	1999	Siberian subtype	BAQ08281
AF527415	Zausaev	Russia	1985	Siberian subtype	AAO43537
MH645617	TBEV-370	Russia	2014	Siberian subtype	AZS54289
MH645615	Lukovka-3	Russia	1986	Siberian subtype	AZS54287
MH645612	3869-03	Russia	2003	Siberian subtype	AZS54284
MG599477	Himalaya-2	China	2013	Himalaya subtype	AWK48862
MG599476	Himalaya-1	China	2013	Himalaya subtype	AWK48861
JN003205	Irkutsk-1861	Russia	2008	Far-Eastern subtype	AEQ77276
AY169390	Primorye-332	Russia	1991	Far-Eastern subtype	AAN87009
HQ201303	Primorye-92	Russia	1992	Far-Eastern subtype	ADX07734
GU121642	Svetlogorie	Russia	2008	Far-Eastern subtype	ADE93003
GQ228395	Primorye-18	Russia	1997	Far-Eastern subtype	ACT32141
FJ997899	Primorye-90	Russia	1990	Far-Eastern subtype	ACR49228
FJ906622	Primirye-89	Russia	1987	Far-Eastern subtype	ACQ99330
FJ402886	Dalnegorsk	Russia	1973	Far-Eastern subtype	ACJ38115
FJ402885	Kavalerovo	Russia	1985	Far-Eastern subtype	ACJ38114
EU816455	Primorye-86	Russia	1986	Far-Eastern subtype	ACF33498
EU816454	Primorye-94	Russia	1994	Far-Eastern subtype	ACF33497
EU816453	Primorye-69	Russia	2000	Far-Eastern subtype	ACF33496
KU761576	Sofjin-1953	Russia	1937	Far-Eastern subtype	AMP18887
KT069219	Primorye-1001	Russia	1958	Far-Eastern subtype	AMP18877
KU761575	Primorye-1285	Russia	1958	Far-Eastern subtype	AMP18886
KU761574	Primorye-1284	Russia	1958	Far-Eastern subtype	AMP18885
KU761573	Primorye-1056	Russia	1958	Far-Eastern subtype	AMP18884
KU761572	Primorye-1035	Russia	1958	Far-Eastern subtype	AMP18883
KU761571	Primorye-1001	Russia	1958	Far-Eastern subtype	AMP18882
KU761570	Primorye-949	Russia	1958	Far-Eastern subtype	AMP18881
KU761569	Primorye-696	Russia	1960	Far-Eastern subtype	AMP18880
KU761568	Primorye-512	Russia	1959	Far-Eastern subtype	AMP18879
KM019546	Tomsk-PT12	Russia	2015	Far-Eastern subtype	AIL33472
KJ914683	Tomsk-M202	Russia	2008	Far-Eastern subtype	AIL33470
KJ914682	Tomsk-PT14	Russia	2008	Far-Eastern subtype	AIL33469
JF819648	SofjinKSY	Russia	1937	Far-Eastern subtype	AEP25267
HQ901367	Primorye-501	Russia	2010	Far-Eastern subtype	AEK94233
HQ901366	Primorye-1153	Russia	2009	Far-Eastern subtype	AEK94232
HM859895	Primorye-2239	Russia	1985	Far-Eastern subtype	ADT80553
HM859894	Primorye-633	Russia	1978	Far-Eastern subtype	ADT80552
EU816452	Primorye-270	Russia	1991	Far-Eastern subtype	ACF33495
EU816451	Primorye-253	Russia	1991	Far-Eastern subtype	ACF33494
EU816450	Primorye-212	Russia	1991	Far-Eastern subtype	ACF33493
DQ989336	205	Russia	1991	Far-Eastern subtype	ABJ74160
MN615728	DXAL-T83	China	2016	Far-Eastern subtype	QLL99544
MN615727	HLB-T74	China	2016	Far-Eastern subtype	QLL99543

MN615726	JL-T75	China	2015	Far-Eastern subtype	QLL99542
KF880805	1230	Russia	2012	Far-Eastern subtype	AHC30239
KF880804	8696	Russia	1986	Far-Eastern subtype	AHC30238
KF880803	9024	Russia	1990	Far-Eastern subtype	AHC30237
JQ825164	Primorye-823	Russia	2000	Far-Eastern subtype	AFP25099
JQ825163	Primorye-750	Russia	1998	Far-Eastern subtype	AFP25098
JQ825162	Primorye-437	Russia	1999	Far-Eastern subtype	AFP25097
JQ825161	Primorye-345	Russia	1999	Far-Eastern subtype	AFP25096
JQ825160	Primorye-320	Russia	1999	Far-Eastern subtype	AFP25095
JQ825159	Primorye-274	Russia	1999	Far-Eastern subtype	AFP25094
JQ825158	Primorye-208	Russia	1991	Far-Eastern subtype	AFP25093
JQ825157	Primorye-202	Russia	1997	Far-Eastern subtype	AFP25092
JQ825156	Primorye-739	Russia	1992	Far-Eastern subtype	AFP25091
JQ825155	Primorye-196	Russia	2000	Far-Eastern subtype	AFP25090
JQ825154	Primorye-52	Russia	1999	Far-Eastern subtype	AFP25089
JQ825153	Primorye-183	Russia	1991	Far-Eastern subtype	AFP25088
JQ825152	Primorye-750	Russia	1999	Far-Eastern subtype	AFP25087
JQ825151	Spassk-72	Russia	1972	Far-Eastern subtype	AFP25086
JQ825150	Primorye-91	Russia	1991	Far-Eastern subtype	AFP25085
JQ825149	Primorye-87	Russia	1987	Far-Eastern subtype	AFP25084
JQ825148	Primorye-823	Russia	1982	Far-Eastern subtype	AFP25083
JQ825147	Shkotovo-94	Russia	1994	Far-Eastern subtype	AFP25082
JQ825146	Kiparis-94	Russia	1994	Far-Eastern subtype	AFP25081
JQ825145	Primorye-895	Russia	2000	Far-Eastern subtype	AFP25080
JQ825144	Primorye-828	Russia	1998	Far-Eastern subtype	AFP25079
JX534167	Xinjiang-01	China	2012	Far-Eastern subtype	AFV48384
JX498940	Sofjin	Clone	2012	Far-Eastern subtype	AFV41132
JX498939	205	Clone	2012	Far-Eastern subtype	AFV41131
AB753012	Oshima 08-As	Japan	2008	Far-Eastern subtype	BAM62429
JQ650523	Senzhang	China	1953	Far-Eastern subtype	AFJ97025
JQ650522	MDJ01	China	2001	Far-Eastern subtype	AFJ97024
GU183380	Kumlinge A52	Finland	1952	Far-Eastern subtype	ADQ00969
JN229223	Sofjin-Ru	Clone	2011	Far-Eastern subtype	AEP20480
JF316708	MDJ03	China	2010	Far-Eastern subtype	ADX87375
JF316707	MDJ02	China	2010	Far-Eastern subtype	ADX87374
AB062063	Oshima 5-10	Japan	1995	Far-Eastern subtype	BAB71943
AB062064	Sofjin-HO	Clone	2000	Far-Eastern subtype	BAB72162
MN115817	1020-69	Russia	1969	Far-Eastern subtype	QDJ94239
KT001073	Lazo MP36	Russia	2014	Far-Eastern subtype	ALA09091
KT001072	Khekhtzir 17-13	Russia	2013	Far-Eastern subtype	ALA09090
KT001071	Khekhtzir 10-13	Russia	2013	Far-Eastern subtype	ALA09089
KT001070	Khekhtzir 9-13	Russia	2013	Far-Eastern subtype	ALA09088
KP869172	Nikolaevsk 855	Russia	1985	Far-Eastern subtype	AKC88490
KP844727	Birobidzhan 1357	Russia	2013	Far-Eastern subtype	AKC88489
KP844726	Birobidzhan 1354	Russia	2013	Far-Eastern subtype	AKC88488
KP844725	Chichagovka 1223	Russia	2012	Far-Eastern subtype	AKC88487
KP844724	Chichagovka 1222	Russia	2012	Far-Eastern subtype	AKC88486
KJ755186	WH2012	China	2012	Far-Eastern subtype	AIG51207
KF951037	4072	Russia	1966	Far-Eastern subtype	AHL20249
EF469661	178-79	Russia	1979	Far-Eastern subtype	ABS00284
MT246197	JL_Jiaohe	China	2019	Far-Eastern subtype	QLH02047
MF374487	Oshima 5.10	Japan	1995	Far-Eastern subtype	AWI66605
