# Supplementary material for: The comparative genomic analysis provides insights into the phylogeny and virulence of tick-borne encephalitis virus vaccine strain Senzhang
Source: PLoS One. 2022 Aug 26;17(8):e0273565. doi: 10.1371/journal.pone.0273565 (PMC9417034; doi:10.1371/journal.pone.0273565)
Supplement: S2 Table — (PDF) [file pone.0273565.s002.pdf]

Position	SNP	Type
243	T > C	Synonymous
345	A > C	Synonymous
375	G > A	Nonsynonymous
679	C > A	Synonymous
684	C > T	Synonymous
708	C > T	Synonymous
771	T > C	Synonymous
831	C > T	Synonymous
834	T > C	Synonymous
877	T > C	Synonymous
882	A > G	Synonymous
888	T > C	Synonymous
930	T > C	Synonymous
939	T > G	Synonymous
987	C > T	Synonymous
1068	T > C	Synonymous
1248	T > C	Synonymous
1471	A > G	Nonsynonymous
1530	C > T	Synonymous
1620	T > C	Synonymous
1716	C > T	Synonymous
1731	C > T	Synonymous
1824	G > A	Synonymous
2109	G > A	Synonymous
2112	T > C	Synonymous
2163	C > T	Synonymous
2286	T > C	Synonymous
2301	C > T	Synonymous
2343	A > G	Synonymous
2347	T > C	Synonymous
2433	C > T	Synonymous
2469	T > C	Synonymous
2628	T > C	Synonymous
2640	G > A	Synonymous
3237	T > C	Synonymous
3630	G > A	Synonymous
3633	T > C	Synonymous
3649	C > T	Synonymous
3729	G > A	Synonymous
3805	T > C	Synonymous
3880	G > A	Nonsynonymous
3981	T > C	Synonymous
3982	T > C	Synonymous
4047	A > G	Synonymous
4062	A > T	Synonymous
4069	G > A	Nonsynonymous
4081	C > T	Nonsynonymous
4084	A > G	Nonsynonymous
4183	T > C	Synonymous
4191	G > T	Synonymous
4209	T > A	Synonymous
4224	A > G	Synonymous
4230	G > T	Synonymous
4233	A > G	Synonymous

4275	T > C	Synonymous
4377	C > T	Synonymous
4380	T > G	Synonymous
4387	T > C	Nonsynonymous
4449	T > A	Synonymous
4683	T > C	Synonymous
4734	C > T	Synonymous
4761	C > T	Synonymous
4830	A > G	Synonymous
4836	C > T	Synonymous
4842	T > C	Synonymous
4860	C > T	Synonymous
4896	C > T	Synonymous
4920	G > C	Synonymous
4971	A > G	Synonymous
4996	T > C	Synonymous
5073	A > G	Synonymous
5076	T > C	Synonymous
5127	C > A	Synonymous
5154	T > C	Synonymous
5271	G > A	Synonymous
5422	A > C	Synonymous
5487	C > T	Synonymous
5604	T > C	Synonymous
5751	G > A	Synonymous
5766	T > C	Synonymous
5862	T > C	Synonymous
6072	A > G	Synonymous
6129	C > T	Synonymous
6267	A > G	Synonymous
6351	T > C	Synonymous
6507	C > T	Synonymous
6771	C > T	Synonymous
6786	T > C	Synonymous
6849	C > T	Synonymous
6956	C > T	Nonsynonymous
6987	A > G	Synonymous
7237	C > T	Synonymous
7255	T > C	Synonymous
7305	A > G	Synonymous
7506	A > T	Synonymous
7536	T > C	Synonymous
7555	T > C	Synonymous
7650	C > T	Synonymous
7704	G > A	Synonymous
7725	G > C	Synonymous
7816	G > A	Nonsynonymous
7974	T > C	Synonymous
8229	T > C	Synonymous
8247	G > A	Synonymous
8340	C > T	Synonymous
8423	A > G	Nonsynonymous
8496	C > T	Synonymous
8562	C > T	Synonymous
8694	T > C	Synonymous
8763	G > A	Synonymous

8775	T > C	Synonymous
8796	T > A	Synonymous
8856	G > A	Synonymous
8859	A > G	Synonymous
8966	G > A	Nonsynonymous
9009	T > C	Synonymous
9118	T > C	Synonymous
9358	T > C	Synonymous
9417	T > C	Synonymous
9526	T > C	Synonymous
9618	C > T	Synonymous
9759	A > G	Synonymous
9804	T > C	Synonymous
9975	A > G	Synonymous
10005	T > C	Synonymous
10022	C > T	Nonsynonymous
10059	T > C	Synonymous
10104	T > C	Synonymous
10229	G > A	Nonsynonymous
10266	G > A	Synonymous
